# Efficacy of virtual block objects in reducing the lung dose in helical tomotherapy planning for cervical oesophageal cancer: a planning study

**DOI:** 10.1186/s13014-018-1012-3

**Published:** 2018-04-04

**Authors:** Makoto Ito, Hidetoshi Shimizu, Takahiro Aoyama, Hiroyuki Tachibana, Natsuo Tomita, Chiyoko Makita, Yutaro Koide, Daiki Kato, Tsuneo Ishiguchi, Takeshi Kodaira

**Affiliations:** 1 Department of Radiation Oncology, Aichi Cancer Center Hospital, Chikusa-ku, Nagoya, Japan; 2 Department of Radiology, Aichi Medical University Hospital, Yazako-Karimata, Nagakute, Aichi 480-1195 Japan

**Keywords:** Cervical oesophageal cancer, Helical tomotherapy, Intensity-modulated radiation therapy, Lung dose, Complete block, Directional block

## Abstract

**Background:**

Intensity-modulated radiotherapy is useful for cervical oesophageal carcinoma (CEC); however, increasing low-dose exposure to the lung may lead to radiation pneumonitis. Nevertheless, an irradiation technique that avoids the lungs has never been examined due to the high difficulty of dose optimization. In this study, we examined the efficacy of helical tomotherapy that can restrict beamlets passing virtual blocks during dose optimization computing (block plan) in reducing the lung dose.

**Methods:**

Fifteen patients with CEC were analysed. The primary/nodal lesion and prophylactic nodal region with adequate margins were defined as the planning target volume (PTV)-60 Gy and PTV-48 Gy, respectively. Nineteen plans per patient were made and compared (total: 285 plans), including non-block and block plans with several shapes and sizes.

**Results:**

The most appropriate block model was semi-circular, 8 cm outside of the tracheal bifurcation, with a significantly lower lung dose compared to that of non-block plans; the mean lung volumes receiving 5 Gy, 10 Gy, 20 Gy, and the mean lung dose were 31.3% vs. 48.0% (*p* <  0.001), 22.4% vs. 39.4% (*p* <  0.001), 13.2% vs. 16.0% (*p* = 0.028), and 7.1 Gy vs. 9.6 Gy (*p* <  0.001), respectively. Both the block and non-block plans were comparable in terms of the homogeneity and conformity indexes of PTV-60 Gy: 0.05 vs. 0.04 (*p* = 0.100) and 0.82 vs. 0.85 (*p* = 0.616), respectively. The maximum dose of the spinal cord planning risk volume increased slightly (49.4 Gy vs. 47.9 Gy, *p* = 0.002). There was no significant difference in the mean doses to the heart and the thyroid gland. Prolongation of the delivery time was less than 1 min (5.6 min vs. 4.9 min, *p* = 0.010).

**Conclusions:**

The block plan for CEC could significantly reduce the lung dose, with acceptable increment in the spinal dose and a slightly prolonged delivery time.

## Background

Cervical oesophageal cancer (CEC) is relatively uncommon, representing 4.4% of all oesophageal cancers [1]. Although surgery has been the primary treatment for CEC, advanced stage is a contraindication for definitive surgery. Additionally, chemoradiotherapy (CRT) is the standard treatment in patients who refuse pharyngo-laryngo-oesophagectomy in order to preserve laryngeal function. A prospective study of CEC treated with 3-dimensional conformal radiotherapy (3DCRT) reported that CRT provides comparable survival to that of surgical resection [2]. Recently, intensity-modulated radiation therapy (IMRT) has gained popularity for CEC [3–5], and provides excellent dose coverage and conformity to the target volume compared to that by 3DCRT [6]. Helical tomotherapy (HT) can achieve higher degrees of dose conformity and homogeneity in the target compared to the traditional IMRT method, owing to a larger number of degrees of freedom in its beam arrangement using a rotating linear accelerator [7–9]. However, our previous study reported that using HT for CEC increases low-dose radiation in the lung compared to that by 3DCRT due to lateral directional beamlets that traverse both sides of the lung during gantry rotations [10]. Although the rate of radiation pneumonitis (RP) in CEC has not been determined, it is a common adverse event in thoracic oesophageal cancer, and low-dose irradiated lung volumes such as V5 and V10 have been reported as important predictive factors for RP [11–13]. An HT function termed “block plan” can restrict beamlets by using a virtual block during computation of dose optimization and may aid in dose reduction. Although only one phantom study has reported this planning technique supposing middle thoracic oesophageal cancer [14], there have been no reports regarding CEC, and the efficiency of this block plan in a clinical setting remains unclear.

Herein, to examine the lung-dose reducing efficacy of the block plan, we compared dosimetric parameters between non-block and block plans with modified conditions.

## Methods

### Patients and simulation

We retrospectively reviewed 20 patients diagnosed with CEC and treated with IMRT between March 2011 and March 2016 at the Aichi Cancer Center Hospital. Five patients were excluded due to exclusion of the lungs in the treatment-planning computed tomography (CT) (*N* = 2) and due to the target volume existing in the superior mandible (*N* = 2) or inferior tracheal bifurcation (*N* = 1) related to the involved lymph node. The remaining 15 patients were included and staged according to the 7th edition of the Union for International Cancer Control [15]. Before acquiring a planning CT scan, upper gastrointestinal endoscopy was performed with iodine staining, and, if necessary, a clip was placed to clarify the tumour extent, especially for superficial lesions. Patients were immobilized in the supine position using a thermoplastic mask which covered from the parietal to the shoulders, and the CT image was obtained with a 2-mm slice thickness. Existing contouring, such as target volumes and organs at risk (OARs), were checked and corrected, and virtual blocks were added as per the protocols described below.

### Virtual block

**Fig. 1:**
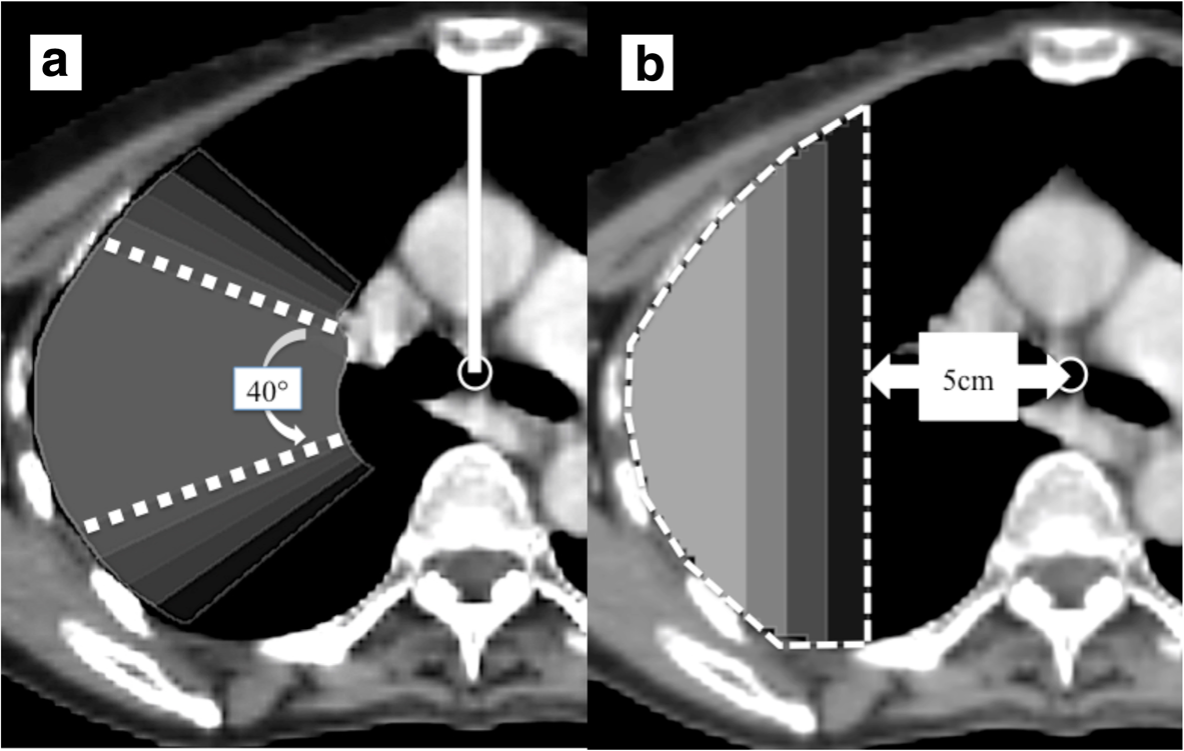
Axial image of virtual blocks. **a** Five fan-shaped blocks in increments of 10 degrees between 40 and 80 degrees, which set the tracheal bifurcation as a central point. **b** Four semi-circular blocks in increments of 1 cm between 5 and 8 cm from the tracheal bifurcation

Two types of virtual blocks were examined: a fan-shaped block and a semi-circular block, contoured using the MIM Maestro™ software (MIM Software, Inc., Cleveland, OH). Firstly, we defined a perpendicular line passing through the tracheal bifurcation as a reference line (Fig. 1a, solid white line). In order to make the fan-shaped block, the tracheal bifurcation was set as a central point (Fig. 1a, black point). For example, for block 40, two lines were drawn to make a fan-shaped block on the right side, with a central angle of 40 degrees (110 degrees and 70 degrees counter-clockwise from the reference line [Fig. 1a, dotted white-lines]). We contoured the right side of the lung between these lines and contoured the left side similarly. For a semi-circular block, the distance from the tracheal bifurcation was measured. For example, for block 5, a 5-cm distance was measured (Fig. 1b, white arrow) and the right lung was contoured outside that region (Fig. 1b, white dot-area); the left side was contoured similarly. We made 5 fan-shaped blocks in increments of 10 degrees between 40 and 80 degrees (Fig. 1a), and delineated 4 semi-circular blocks in increments of 1 cm between 5 and 8 cm (Fig. 1b). However, a portion of the block within 1 cm of the planning target volume (PTV) was removed in order to avoid beam interference.

### Target and organs at risk setting

The gross tumour volume of the primary lesion (GTVprimary) was defined by endoscopy and diagnostic imaging (barium contrast study, CT, or positron emission tomography-CT). The clinical target volume of the primary lesion (CTVprimary) was defined as the GTVprimary with 2 cm of cranio-caudal margin on the oesophageal wall and a 0.5-cm margin in the lateral direction. The CTV of the nodal lesion (CTVnode) was defined as the involved lymph node, with a 0.5-cm margin in every direction. The CTV for the prophylactic area (CTVprophylactic) ranged from the level III cervical node to the upper mediastinal node, including 101–107 lymph nodes (Japanese Classification of Esophageal Cancer 11^th^edition) [16]. The PTV (PTVprimary/node/prophylactic) was defined as CTVprimary/node/prophylactic with a 0.5-cm margin in all directions. The OARs included the lung, heart, thyroid, and spinal cord. For the spinal cord, a planning risk volume (PRV) with a 5-mm margin was created.

### Treatment system and planning optimization

A Helical Tomotherapy Hi-Art treatment system (Accuray, Madison, WI) was used for delivering IMRT treatments. Planning Stations (Accuray, Madison, WI) were used to optimize the IMRT dose prescription. The planning parameter values were as follows: modulation factor: 2.2, pitch: 0.43, and field width: 2.5 cm for all cases [17]. The superposition algorithm and a calculation matrix of 2 mm for all directions were used. Virtual blocks were used in two modes: complete mode, which restricts beamlets that pass the block, and directional mode, which permits beamlets that reach the block after passing through the PTVs. For the PTVprimary and PTVnode, 60 Gy in 30 fractions were delivered, while 48 Gy in 30 fractions were delivered for the PTVprophylactic, using a simultaneous integrated boost technique. The goal was to cover 95% of each PTV with the prescribed dose (D95%). Specifically, dose constraints were defined as follows: D98% > 54.0 Gy (90%), D95% > 58.8 Gy (98%), D50% < 64.2 Gy (107%), D10% < 69.0 Gy (115%), and D2% < 72 Gy (120%) for the PTVprimary and PTVnode; D98% > 43.8 Gy (73%), D95% > 46.8 Gy (78%), D50% < 55.8 Gy (93%), and D2% < 64.2 Gy (107%) for the PTVprophylactic; Dmax < 52 Gy, D2% < 50 Gy for the PRV of the spinal cord; and Dmax < 75 Gy (125%) for the whole body. Nineteen plans (a non-block plan and block plans using the above-mentioned two modes for 5 fan-shaped and 4 semi-circular blocks) per patient were designed, and a total of 285 plans were compared. The same radiation oncologist that routinely makes the inverse planning performed all planning optimizations to prevent technical biases. The plans that did not meet the constraints were excluded from the analysis.

### Scoring and additional analyses

Each block plan was scored to evaluate its efficacy. Based on previous studies on RP, the compliance criteria of the lung volume receiving 5 Gy (V5), 10 Gy (V10), 20 Gy (V20), and the absolute volume of the lung spared from 5 Gy (VS5) were defined as 55%, 37%, 25%, and 1500 mL, respectively [11, 12, 18]. Each plan was scored (0–5) according to the grade of reduction in the lung dose (Table 1). The number of cases with plan approval (0–15) was added to this score to obtain a total block score (0–35). Three high-scoring blockage modes with scores > 30 were analysed by one-way analysis of variance. The indices were calculated as follows:

1 $$ \cdot \mathrm{conformity}\ \mathrm{index}\ \left(\mathrm{CI}\right)=\mathrm{Paddick}\ \mathrm{CI}=\left(\mathrm{TV}\mathrm{PIV}\right)2/\left(\mathrm{TV}\times \mathrm{PIV}\right), $$

**Table 1:** Definition of the scores

	clear the criteria	reduce the criteria 20%	reduce the criteria 30%	reduce the criteria 40%	reduce the criteria 50%
Score	1	2	3	4	5
V5 (%)	44< LV ≦55	38.5< LV ≦44	33< LV ≦38.5	27.5< LV ≦33	LV ≦27.5
V10 (%)	29.6< LV ≦37	25.9< LV ≦29.6	22.2< LV ≦25.9	18.5< LV ≦22.2	LV ≦18.5
V20 (%)	20< LV ≦25	17.5< LV ≦20	15< LV ≦17.5	12.5< LV ≦15	LV ≦12.5
VS5 (mL)	1800> LV ≧1500	1950> LV ≧1800	2100> LV ≧1950	2250> LV ≧2100	LV ≧2250

*V5* lung volume receiving 5 Gy, *V10* lung volume receiving 10 Gy,*V20* lung volume receiving 20 Gy, *VS5* normal pulmonary volume of less than 5 Gy, *LV* average lung volume

[19].

where TVPIV: PTVprimary/node volume covered by the prescription isodose.

TV: PTVprimary/node volume.

PIV: prescription isodose

2 $$ \cdot \mathrm{homogeneity}\ \mathrm{index}\ \left(\mathrm{HI}\right)=\left(\mathrm{D}2\%\hbox{-} \mathrm{D}98\%\right)/\mathrm{D}50\%, $$

[20].

where D2%, D98%, and D50%: minimum doses in 2%, 98%, and 50% of the PTVprimary/node volume, respectively.

Delivery time was defined as the beam-on-time displayed on Planning Stations after the final calculation.

Statistical analysis was performed with EZR version 1.33 (Saitama Medical Center, Jichi Medical University, Saitama, Japan), which is based on R and R commander [21].

## Results

### Patient characteristics

The patient characteristics are listed in Table 2. Since elderly people and women were included, there were several patients with a low lung volume: the median volume was 3389 mL (range: 2319–4448 mL). The primary tumours tended to be advance (T3–4), and 2 cases showed thyroidal invasion. The patients showed 1–6 lymph nodal invasions between the supraclavicular area and the tracheal bifurcation, except in 1 case.

**Table 2:** Patient characteristics

No. of patients	15
Age (years)	
median	64
range	54–81
Sex	
Male	10
Female	5
Tumour size (cm)	
median	4
range	2–10
T - category	
1	0
2	3
3	9
4	3
N - category	
0	1
1	7
2	7
M - category	
0	15
1	0
UICC stage	
IIA	1
IIB	2
IIIA	6
IIIB	3
IIIC	3
Lung volume (mL)	
median	3389
range	2319–4448

### Presentation of a sample case

**Fig. 2:**
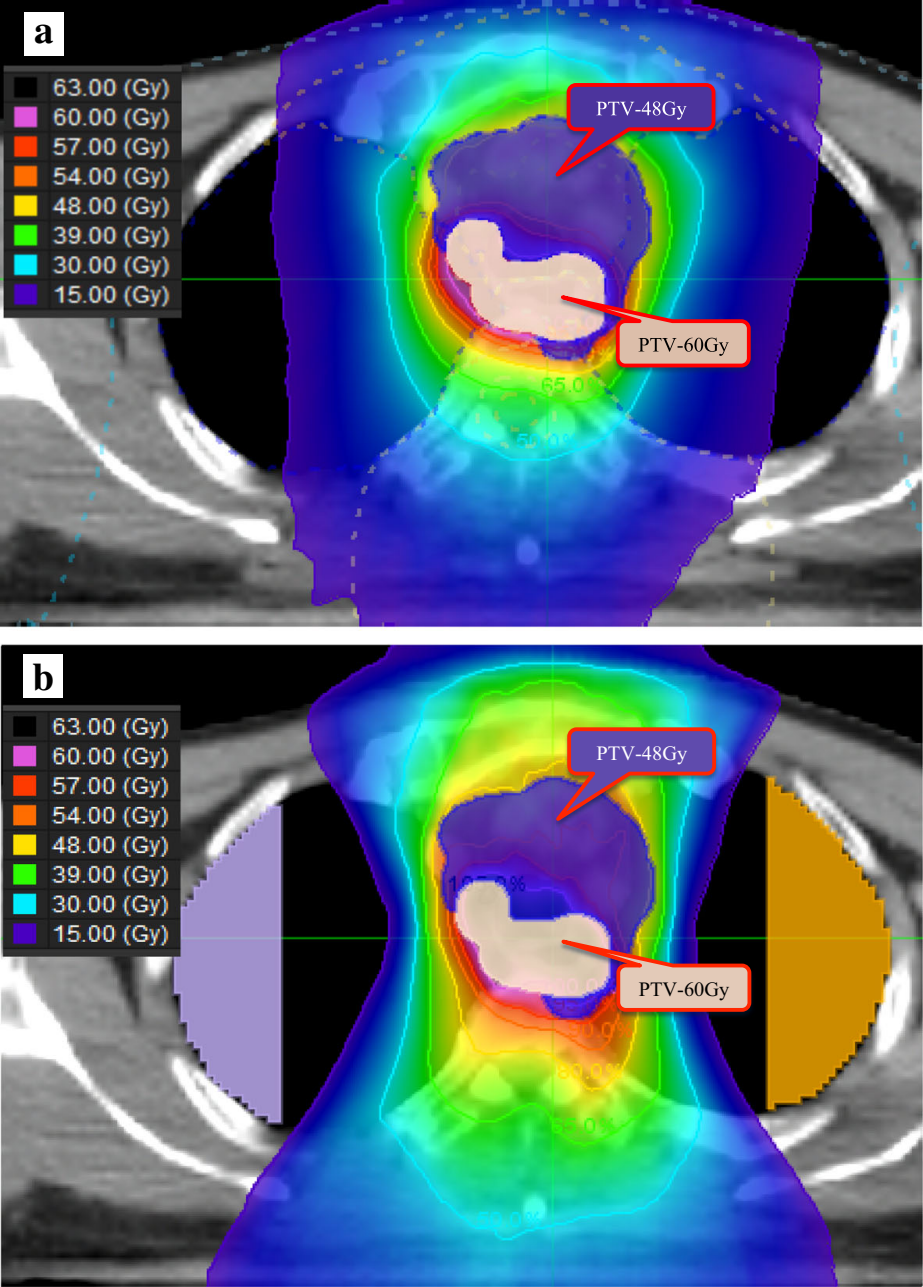
Comparison of the dose distribution of non-block and block planning. **a** Non-block plan; (**b**) semi-circular plan for block 7. Each plan was made using the same target volume pertaining to a single patient. The dose gradient of block 7 was steeper than that of the non-block plan. Block 7: semi-circular virtual block that contoured the lungs outside a distance of 7 cm from the tracheal bifurcation

**Fig. 3:**
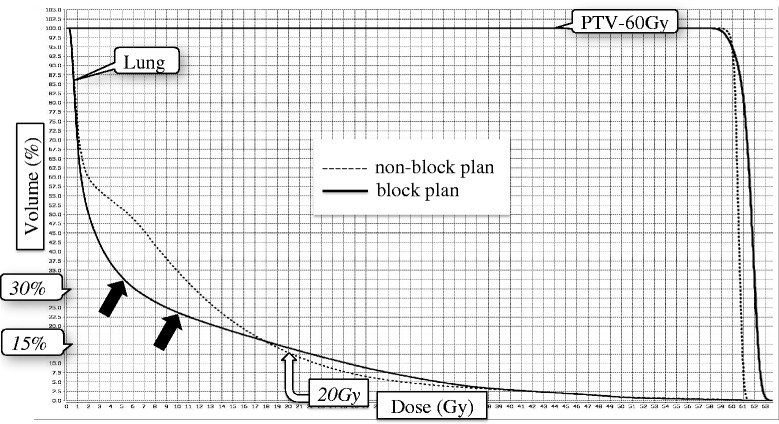
Comparison of the dose-volume histogram of non-block and block planning. Although the V20 values of the lungs were comparable, with approximately 15% between the plans, V5 and V10 of the block plans were much lower than those in the non-block plan (black arrow).V20, the lung volume receiving 20 Gy; V5, the lung volume receiving 5 Gy; V10, the lung volume receiving 10 Gy

Two dose distributions of a sample case are shown in Fig. 2. In the non-block plan, low-dose radiation spread widely in the lung (Fig. 2a). When we used the appropriate block, the dose gradient was very steep, and the lung dose was reduced (Fig. 2b). Dose-volume histograms for the lung and PTV primary/node are shown in Fig. 3. There was no significant difference in V20 of the lungs (approximately 15% variation) between the plans; however, V5 or V10 was high in the non-block plan. In the block plan, we could reduce low-dose radiation in the lung while maintaining dose coverage and homogeneity to the PTVprimary/node.

### Lung dose and score for each block plan

The mean values of the lung dose and score for each fan-shaped block are listed in Table 3. In the non-block plan group (controls), the plans satisfied the dose constraints in all 15 cases, with a score of 15. The V5 score, however, was 1, since V5 fell below the compliance criteria in this group. In addition, this group was scored as 0, 3, and 1 for V10, V20, and VS5, respectively. Thus, the non-block plan group scored 20 in total. As none of the fan-shaped blocks in the complete mode plan were defined as approved plans, they could not be scored. In the directional mode, when using a block with a central angle of 50 or 60 degrees, the compliance rate of the restriction and the reduction in lung dose were the most well-balanced. When the central angle was 70 degrees or more, the constraints could not be met. The highest score of a fan-shaped block plan was 29. In plans using semi-circular blocks (Table 3), the blocks placed at a distance of ≥7 cm from the tracheal bifurcation were preferred. The highest score was 32, for a block 7 directional mode plan, followed by a score of 31 for directional mode and complete mode block 8 plans.

**Table 3:** Comparison between plans using a fan-shaped block and those using a semi-circular block

		Block mode	Number of approved plan		Lung (mean ± SD)			Score
				V5 (%)	V10 (%)	V20 (%)	VS5 (mL)	
Non-block			15/15	48.0 ± 7.9	39.4 ± 6.9	16.0 ± 3.0	1747 ± 522	20
fan-shaped block	Block 40	C	0/15	NA	NA	NA	NA	0
		D	15/15	38.7 ± 6.4	27.0 ± 4.8	12.9 ± 2.9	2049 ± 527	26
	Block 50	C	0/15	NA	NA	NA	NA	0
		D	15/15	36.5 ± 5.9	25.4 ± 4.4	12.6 ± 2.7	2124 ± 535	29
	Block 60	C	0/15	NA	NA	NA	NA	0
		D	15/15	34.4 ± 5.7	23.9 ± 4.3	13.0 ± 3.3	2197 ± 560	29
	Block 70	C	0/15	NA	NA	NA	NA	0
		D	11/15	33.5 ± 5.9	23.1 ± 4.4	12.3 ± 2.8	2223 ± 585	26
	Block 80	C	0/15	NA	NA	NA	NA	0
		D	3/15	36.5 ± 6.4	25.5 ± 4.9	14.1 ± 4.1	2052 ± 291	16
semi-circular block	Block 5	C	0/15	NA	NA	NA	NA	0
		D	1/15	29.7	20.3	12.4	2434	19
	Block 6	C	0/15	NA	NA	NA	NA	0
		D	6/15	29.1 ± 3.3	19.7 ± 3.2	12.0 ± 3.1	2396 ± 288	24
	Block 7	C	6/15	22.0 ± 3.0	17.0 ± 2.3	11.4 ± 1.6	2391 ± 506	26
		D	14/15	29.2 ± 3.9	20.6 ± 3.5	12.1 ± 2.7	2308 ± 525	32
	Block 8	C	13/15	26.4 ± 4.4	20.6 ± 3.6	13.3 ± 2.9	2454 ± 586	31
		D	15/15	31.3 ± 4.8	22.4 ± 3.9	13.2 ± 3.0	2299 ± 574	31

*V5* lung volume receiving 5 Gy, *V10* lung volume receiving 10 Gy, *V20* lung volume receiving 20 Gy, *VS5* normal pulmonary volume of less than 5 Gy, *C* complete block mode, *D* directional block mode, *NA* not available

### Dosimetric parameter comparison among selected blocks

We further analysed plans with scores > 30. The plans using directional mode block 7 and block 8 were selected and compared with the non-block plan of the control group. As Table 4 shows, the lung dose was significantly reduced in all block plans. Without the block, there were 3, 10, and 5 cases (among 15), in which the lung volume exceeded the compliance criteria for V5, V10, and VS5, respectively. Alternatively, by using block 7 or 8, we were able to meet the criteria in all cases. Between the plan using the directional mode for block 8 and the non-block plan, there were no differences in the CI, HI, and body max dose, while compared to the other block plans, the CI of these plans decreased, and the HI and body max dose increased. By using a block, the maximum dose of the spinal cord PRV increased significantly but slightly: 47.9 to 49.4 Gy when using a block 8 in the directional mode. There was no difference in the average doses to the heart and thyroid among these plans. Although the dose delivery time was prolonged in all block plans, the increase in time was within 1 min for block 8 with directional mode.

**Table 4:** Dosimetric parameter comparison among a selected series of blockage modes using a semi-circular block

		Non-block (control group)	Block 7 (D)	*p*	Block 8 (C)	*p*	Block 8 (D)	*p*
Lung	V5 (%)	48.0	29.2	< 0.001	26.4	< 0.001	31.3	< 0.001
	V10 (%)	39.4	20.6	< 0.001	20.6	< 0.001	22.4	< 0.001
	V20 (%)	16.0	12.1	0.002	13.3	0.046	13.2	0.028
	VS5 (mL)	1747	2308	0.023	2454	0.004	2299	0.023
	mean dose (Gy)	9.6	6.8	< 0.001	7.0	< 0.001	7.1	< 0.001
Conformity Index		0.85	0.77	0.012	0.79	0.131	0.82	0.616
Homogeneity Index		0.04	0.07	0.001	0.07	0.002	0.05	0.100
Spinal cord PRV max dose (Gy)		47.9	49.7	< 0.001	50.2	< 0.001	49.4	0.002
Body max dose (Gy)		63.3	64.7	0.005	65.2	< 0.001	63.9	0.317
Heart mean dose (Gy)		6.2	7.4	0.564	7.2	0.719	7.0	0.811
Thyroid mean dose (Gy)		53.6	54.0	0.964	55.4	0.315	54.0	0.968
Delivery time (min)		4.9	6.0	< 0.001	6.2	< 0.001	5.6	0.010

*V5* lung volume receiving 5 Gy, *V10* lung volume receiving 10 Gy, *V20* lung volume receiving 20 Gy, *VS5* normal pulmonary volume of less than 5 Gy, *C* complete block mode, *D* directional block mode, *PRV* planning risk volume

## Discussion

In recent years, IMRT has been used for oesophageal cancer [22, 23]. The fixed-field IMRT technique is more suitable for thoracic oesophageal cancer rather than HT, considering the increase in lung and heart doses. Martin et al. [24] reported that the lung dose for the fixed-field IMRT with conformal arc technique was significantly reduced compared to HT for middle/distal oesophageal cancer, with acceptable PTV coverage; the mean V10 (66.2%), V15 (34.8%), and mean lung dose (MLD) (26.3%) of HT were improved to 40.3%, 25.2%, and 21.2%, respectively. As for CEC, there are several reports that HT or volumetric-modulated arc therapy (VMAT) are preferable than fixed-field IMRT [8, 25–27]. Yin et al. [25] compared VMAT plans with conventional IMRT plans for 5 patients with CEC. According to their report, the CI was significantly higher in VMAT (single arc: 0.78, double arc: 0.8) than in conventional IMRT (5, 7, and 9 fields: 0.62, 0.66, 0.73, respectively), and the lung V30 was lower in VMAT (single arc: 12.52, double arc: 12.29) than in conventional IMRT (7 and 9 fields: 14.35 and 14.81, respectively). The difference in the preferred irradiation method between thoracic oesophageal cancer and CEC might be based on the difference in the range of the elective nodal irradiation area. Hirano et al. [28] reported that the incidences of cervical lymph nodal and upper mediastinal metastasis in 21 patients with CEC were 85.7% (especially wherein 43% were levels II–III) and 33.3%, respectively, and asserted that neck lymph node (levels II–IV) and upper mediastinal dissection was crucial to improve the cure rate. Accordingly, many institutions have set the prophylactic area covered from level II or III of the cervical nodes to the subcarinal nodes of the mediastinum [5, 29], and the extensive exposure field where the body thickness differs thus causes difficulties in planning for CEC. Although HT could provide excellent target volume coverage and conformity for CEC, the pulmonary dose increment is a problem, and we therefore examined a novel planning method in this study. The block plan is different from the standard plan in the optimization process during the treatment planning only. The standard plan uses all beamlets in the optimization process. In contrast, the block plan excludes all or some of beamlets passing through the block. As a result, the block plan can provide a protective effect for normal tissue by multi-leaf collimators, effectively.

We aimed to establish a universal planning method, considering 3 points. Firstly, virtual blocks based on the tracheal bifurcation (which can be replicated easily) were defined. Secondly, 15 patients of both sexes and various ages, with varied tumour sizes and lung volumes, were included (Table 2), and the targets and OARs were described by a unified protocol. Finally, a total of 285 block plans were compared and the type, size, and mode of the final block plan were optimized. As a result, we were able to define an optimal block plan with a standardized study design (most well-balanced: block 8 directional mode plan), which was a significant achievement.

In comparing the efficacy between the virtual blocks objectively, each block plan was scored.

In the non-block plan group, all 15 cases satisfied the dose constraints, with good CI (0.85) and HI (0.04), owing to the unconstrained irradiation beam angle. However, low-dose radiation spread widely in the lung, lowering the score (20 out of 35), and the mean V10 value was unable to clear the compliance criteria. Although we delineated the lung as an OAR and imposed strong restrictions, the effect of the lateral directional beamlets, which traverse both sides of the lung, was not suppressed. The fan-shaped block used in the directional mode, on the other hand, led to improved scores (Table 3). Since the irradiation beamlets would be restricted to protect the fan-shaped area in the lungs, when the central angle was larger, a larger lung volume would be protected. However, when the central angle was 70 degrees or more, the dose constraints of the spinal cord PRV were not met, because beamlets were directed dorsoventrally. None of the plans with complete mode groups for a fan-shaped block were defined as approved plans. Although the block sections overlapping with the PTV plus a 1-cm area were removed, we were not able to plan optimization of the supraclavicular prophylactic area adequately. Semi-circular blocks were more effective than fan-shaped blocks in reducing the lung dose, and the dose reduction improved with block size. In cases with underlying diseases like interstitial pneumonitis, it might be preferable to use block 7 for directional mode or block 8 for complete mode, in order to reduce the lung dose as much as possible. However, evaluation of the plan quality in the context of all parameters is required; it is important that high doses are not prescribed out of the PTV for a patient, and that the dose in the PTV is homogeneous. By using a block, the maximum dose of the spinal cord PRV increased slightly but significantly: for example, from 47.9 to 49.4 Gy when using a block 8 in the directional mode. It must be recognized that the option of future re-irradiation may be limited due to this slight increase. Therefore, when using a block, a lower spine dose plan should be considered, if all other parameters being equal. Lowering the irradiation time is also important in reducing the patient burden. Although prolongation of the irradiation time is unavoidable due to beamlet restriction, this time should be shortened as much as possible. Therefore, we consider that the best plan was the directional mode plan for block 8, which could significantly reduce the lung dose without adversely affecting other dosimetric parameters (e.g. CI and HI), while increasing the delivery time by under 1 min. The most important contribution of our study is the universal block planning method for CEC. At first, we considered that the block size may need patient-specific modification, but the block 8 plan for the directional mode showed favourable results in all cases, across a large range of lung volumes (2319–4448 mL). As shown in Table 3, the reduction in the lung dose was inadequate or did not clear the dose constraints when an unsuitable block was used. Since changes in the size of the block or the mode of the plan would require re-optimization, applying an optimized plan without changes could shorten planning times remarkably.

Dosimetric parameters of lung V5, V10, and VS5, which were used as compliance criteria in our study, are important factors associated with RP. Tanabe et al. [12] studied 86 patients with locally advanced oesophageal cancer treated with definitive CRT (radiation dose: 50.4 or 59.4 Gy). Patients with Grade 0–1 RP showed significantly lower V5 and V10 values for the whole lung compared to those with Grade 2–5 RP. The proposed plan led to advantageous V5 (< 55%) and V10 (< 37%) values, and conferred PTV conformity. Tsujino et al. [18] reported that 14 (11.4%) of 122 patients with locally advanced non–small-cell lung cancer treated with concurrent CRT developed RP greater than or equal to Grade 3, and revealed that VS5 < 1500 cm^3^ was a significant risk factor for RP. We consider that using a block plan and meeting their criteria might reduce the RP risk. On the other hand, the non-block plan also showed low V20 and MLD values (16.0% and 9.6 Gy, respectively), since we analysed typical CEC patients with the target’s inferior border set to the tracheal bifurcation. Chang et al. [14] reported a phantom study supposing middle thoracic oesophageal cancer, and reported a substantial reduction in the lung dose using a fan-shaped complete block compared to a non-block design (reductions in V20, V15, V10, V5, and MLD of 6.3–8.6%, 16–23%, 42–57%, 42–66%, and 5.2 Gy–7.5 Gy, respectively). Since thoracic oesophageal invasion or a skip lesion in the thoracic oesophagus is often observed in CEC, the target’s inferior border to the middle or lower thoracic oesophagus is planned to be expanded. When performing such an extended irradiation, we consider that a block plan can further reduce the RP risk. In addition, we are now able to use a TomoDirect mode that allows delivery of radiation at pre-established discrete angles with a fixed gantry [30]. Although the efficacy of the TomoDirect mode for oesophageal cancer has not yet been determined, Murai et al. [31] reported its usefulness in lung cancer compared to the Tomohelical mode (lung V5: 30 ± 3% vs. 43 ± 3%, *P* = 0.005; *n* = 18). Thus, combining a TomoDirect mode and a block plan may result in a higher quality plan.

The most important limitation of our study is the lack of verification. We confirm that the passing rate of gamma analysis (2 mm /2%, threshold 20%) for the 6 block plans that were actually used in the clinical treatment by the ArcCHECK quality assurance phantom (Sun Nuclear, Melbourne, FL) was more than 90%. However, where the study plans were not used in practice, verification was not performed. The HT achieves high-dose conformity according to accurate motion of the multileaf collimator. Since beamlets are steeply restricted according to a gantry angle, the block plan might be largely affected by setup error compared with the non-block plan. It is thus essential to clarify the relation between positioning error and dose coverage of the PTV in future studies. Another limitation concerns the comparison of block plans. The scoring system used was adapted for comparing a large number of plans rapidly and was not based on established evidence. In addition, although the block 8 directional mode plan reduced the lung dose significantly compared with non-block plans, it was not always statistically superior compared with other block plans. Although more superior block types than those examined here may exist, our technique might be an effective alternative option, especially in institutions where block plans are not used.

## Conclusions

When helical irradiation is performed in the usual way for CEC, compliance criteria of the lung dose are often exceeded. To reduce the RP risk, a novel planning method which can be applied universally, is required. The block plan using HT can significantly reduce the lung dose, with acceptable increment of a spinal dose and a slightly prolonged irradiation time. Use of a semi-circular block at a distance of 8 cm from the bifurcation with directional mode is recommended based on our analysis.

## Abbreviations

3DCRT: 3-dimensional conformal radiotherapy
CEC: Cervical oesophageal carcinoma
CI: Conformity index
CRT: Chemoradiotherapy
CT: Computed tomography
CTV: Clinical target volume
GTV: Gross tumour volume
HI: Homogeneity index
HT: Helical tomotherapy
IMRT: Intensity-modulated radiation therapy
MLD: Mean lung dose
OAR: Organ at risk
PRV: Planning risk volume
PTV: Planning target volume
RP: Radiation pneumonitis
VMAT: Volumetric-modulated arc therapy
